# A Review of Prestressed Fibre-Reinforced Polymer Matrix Composites

**DOI:** 10.3390/polym14010060

**Published:** 2021-12-24

**Authors:** Raphael Olabanji Ogunleye, Sona Rusnakova

**Affiliations:** Department of Production Engineering, Faculty of Technology, Tomas Bata University in Zlín, Vavrečkova 275, 760 01 Zlin, Czech Republic; rusnakova@utb.cz

**Keywords:** polymer composite, fibre-prestressing, residual stresses

## Abstract

This review examines various studies on reducing tensile stresses generated in a polymer matrix composite without increasing the mass or dimension of the material. The sources of residual stresses and their impacts on the developed composite were identified, and the different techniques used in limiting residual stresses were also discussed. Furthermore, the review elaborates on fibre-prestressing techniques based on elastically (EPPMC) and viscoelastically (VPPMC) prestressed polymer matrix composites, while advantages and limitations associated with EPPMC and VPPMC methods are also explained. The report shows that tensile residual stresses are induced in a polymer matrix composite during production as a result of unequal expansion, moisture absorption and chemical shrinkage; their manifestations have detrimental effects on the mechanical properties of the polymer composite. Both EPPMC and VPPMC have great influence in reducing residual stresses in the polymer matrix and thereby improving the mechanical properties of composite materials. The reports from this study provide some basis for selecting a suitable technique for prestressing as well as measuring residual stresses in composite materials.

## 1. Introduction

Composite materials are developed by combining materials offering unique properties that cannot be achieved individually by the constituent materials. Because of their excellent strength to weight ratio, they have increasingly been used as engineering materials for many applications, that include automotive and aerospace parts, construction materials, electrical parts and other consumer products [1]. The growth of composite materials for extended use, particularly in the aerospace and automobile industries, has necessitated continuous research for developing improved composites with excellent mechanical properties [2]. The constituents of composites are classified into matrix and reinforcement. While the matrix can be made of polymers, metals or ceramics, reinforcement includes fibres, whiskers and particulate fillers that exist in natural (lignocellulose, animal fibres and minerals) or synthetic form (carbon, aramid, boron, nylon, polyethylene) [3].

To produce a fibre-reinforced polymer composite, fibres of various configurations and stiffness are embedded into a polymer matrix of lower stiffness. While the fibre is responsible for carrying the load and offering much needed strength and stiffness, the polymer matrix is responsible for the mobility of the load to other parts of the fibre by providing the required binding forces [3]. The matrices also prevent the reinforced fibres from absorbing moisture, propagating micro-cracking due to microbial and chemical attacks. Polymer matrices can be either thermoplastic or thermoset. Thermoplastic polymers undergo a physical change when heated and can be softened and reformed. Some thermoplastics commonly used in composites include polyetheretherketone (PEEK), polyetherketoneketone (PEKK), polyphenylene sulphide, polyethylenimine (PEI) and polycarbonates (PC) [4]. On the other hand, thermoset polymers undergo a chemical change in the presence of a crosslinking agent (hardener) to form a three-dimensional structure. Thermosets include epoxies, phenolics, polyimides, polyester, vinyl ester, bismaleimide, melamine and silicone [4]. Thermoplastic composites offer great potential due to less processing time. However, significant drawbacks, which include a high processing temperature (the polymers must be heated to melting point to incorporate the fibre), a high tendency for the buckling of fibres in the polymer matrices, and solvent and fluid resistance features, have reduced their adoption as a substitute for thermoset in fibre-reinforced polymer composites [5].

Both tensile and compressive stresses are generated in polymer composite structures during the manufacturing process, and their occurrence in a composite can result from differences in physical and mechanical properties, a contraction of the polymer matrices before curing, manufacturing techniques and moisture absorption from the environment [6]. However, while compressive stresses have positive influences on the mechanical properties of the composite material, the tensile stresses have a detrimental effect on the manufactured polymer composite by causing distortion, dimensional instability and matrix cracking [7]. The residual stresses referred to in this study are the tensile residual stresses, and many techniques have reportedly been used in the literature to lessen the residual stresses in fibre-reinforced polymer (FRP) composites. Fibre-prestressing offers a low-cost means of reducing the influence of tensile residual stresses, thereby improving the mechanical properties without increasing the mass or dimension of the composite [8]. It is important to know that fibre-prestressing can only reduce residual stress in polymer composites if applied to a certain level [9]. Therefore, there has been continuous research on improving fibre-prestressing techniques.

This review explores the state-of-the-art fibre-prestressing techniques used in reducing residual stress in FRP composites. Moreover, the mechanism and methods used for improving the mechanical properties of FRP composites are examined.

## 2. Source of Residual Stresses in a Composite

In the absence of external forces, residual stresses are forms of stress that bring about the deformation of a composite material [7]. There are two primary sources of residual stresses in a composite material; (i) residual stresses generated due to differences in deformation-related properties of the materials, and (ii) production processes that induce residual stresses [9]. Moisture absorption, the chemical shrinkage of the matrix and a variation in the coefficient of thermal expansion induce residual stresses in polymer composites. Researchers have also shown that residual stresses can initiate failure in a composite material due to matrix cracking [10]. The influence of matrix cracking can lead to a dangerous failure, especially when the material is under loading conditions. Both compressive and tensile residual stresses are generated in composite materials. However, while compressive residual stresses help prevent cracks from spreading throughout the matrices, tensile residual stresses assist in the opening of micro-cracks in the polymer matrix [11]. Other defects such as fibre waviness, delamination, warping and dimensional imbalance are also due to residual stresses [12].

**Figure 1:**
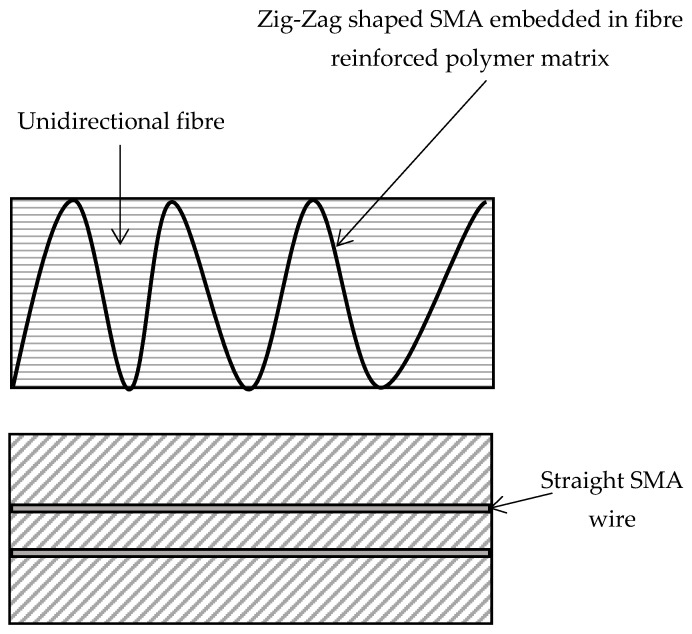
Integration of SMA into a Fibre-Reinforced Polymer Composite.

Many techniques have reportedly been used to reduce the effect of residual stresses in polymer composites. Process optimisation of cure cycle parameters such as dwell time, dwell temperature, dwell cycle and cooling rate can minimise residual stress during production [13,14,15]. Shape memory alloys (SMAs) also reduce stress concentration in FRP matrices. SMAs are novel metallic materials that can undergo reversible solid to solid change in a phase when subjected to thermal or mechanical loading that can cause sizeable inelastic strain [16]. They are generally available in the form of a wire with a diameter below 200 µm [17] and can be embedded in various configurations, as shown in Figure 1. SMAs offer other vital properties such as impact damage control, crack closure and shape morphing. Nevertheless, SMAs induce fibre misalignment and can create an uneven stress distribution in a composite [17].

Similarly, the electron beam curing technique composite can also reduce stress concentration [18]. The technique involves generating free radicals from a high-energy electron accelerator that propagates the polymerisation and crosslinking reaction at room temperature. Since the curing temperature is kept below the traditional thermal curing process temperature, the residual stress generated using an electron beam will be lowered [19]. Moreover, high processing electron beam curing results in an increased production rate and low shrinkage compared to that of conventional thermally cured composites [20]. To utilise the electron beam curing process, cationic initiators are required. Hence, the initiators—that are primarily Lewis or Bronsted acids—react when exposed to high energy electron irradiation. However, a high initiator concentration can lead to a thermal degradation of the polymer matrix, while excess irradiation can damage the fibre strength [21].

Despite their positive influences, electron beam cured composites cannot replicate some of the characteristics of thermally cured composites (high fracture toughness and inter-laminar shear strength) [20]. Similarly, expanding monomers have reportedly been used as anti-shrinkage in composite materials [22]. The polymerisation reaction of the polymer matrices results in a volumetric shrinkage of the matrices during the curing stage. The addition of the expanding polymer gives rise to volumetric expansion, thereby reducing volumetric shrinkage and residual stress [23,24]. The expanding monomers can also reduce the modulus and initiate the transverse cracking of composite materials [9]. Compared to the techniques mentioned above concerning the cost and simplicity, fibre-prestressing is relatively more acceptable.

## 3. Fibre-Prestressing Technique

There has been growing interest in improving the mechanical performance of FRP composites by minimising the induced stress without increasing the mass or dimension of the composite [8]. Since improving the composite performance increases the production cost, improving the production technique of composites through fibre-prestressing is a possible way of mitigating the cost [9]. Fibre-prestressing has been discovered by researchers as a productive method of reducing the effect of residual stresses in composite materials during production [25,26,27].

Fibre-prestressing can be achieved elastically or viscoelastically. The elastically prestressed polymer matrix composite (EPPMC) is achieved by subjecting the fibre material to the applied load and maintaining the load throughout the curing cycle of the polymer composite [28,29]. The load is released after the cooling and solidification of the composite at ambient temperature. Subsequently, the prestressed fibres tend to return to their initial length, generating compressive stresses in the matrix. These compressive stresses reduce the detrimental effect of the tensile residual stresses induced during the curing process [29]. In addition, the viscoelastically prestressed polymer matrix composite (VPPMC) is formed by subjecting polymeric fibres to a tension load, which introduces creep stress, and then removing the load before moulding the fibre to the polymer matrix. After the curing stage, prestressed fibres tend to return to their initial length, thereby creating compressive stresses that counterbalance the effect of induced tensile residual stresses [28]. The advantages and shortcomings of the two prestressing techniques are discussed further in the next section.

### 3.1. Elastically Prestressed Polymer Matrix Composite (EPPMC)

The fibres are prestressed by subjecting them to a force lower than the elastic limit of the fibre and maintaining this force throughout the curing cycle of the composite. After curing, the force is removed, and the fibres tend to return to their original states. Compressive stresses are developed within the solidified matrix due to the bonding of the prestressed fibre with the polymer matrix [30]. Various methods that have reportedly been used to elastically prestressed fibres are discussed in the next section.

#### 3.1.1. Deadweight Method

**Figure 2:**
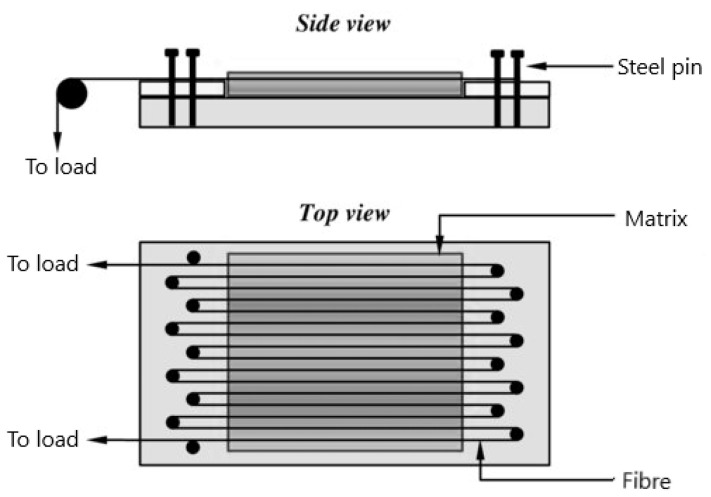
The deadweight prestressing method used by Jorge, Marques and De Castro [6].

The deadweight prestressing method (Figure 2) was first presented by Jorge, Marques and De Castro [31]. They studied the influence of prestressing on the mechanical behaviour of a unidirectional polyester composite using E-glass fibre. The deadweight rig consists of steel pins arranged on a roving boundary, with the distance between the adjacent pin approximately equal to 2 mm. The fibres were rolled around the steel pins, and the two ends were subject to a load. The resin was applied, and the composite was cured at ambient temperature in an oven. Al-Dulaimy, Al-hassany and Shakir [32] utilised a similar prestressing technique using E-glass fibre and epoxy resin. The result obtained shows an increase in stiffness and a percentage elongation at break for the prestressed composite. Some of the shortcomings of using the deadweight method include the inability to obtain a uniform distribution of fibres in the polymer matrix and a fibre fracture tendency in the steel pin area when subjected to a bending force [33].

#### 3.1.2. V-Slot Fastening Method

**Figure 3:**
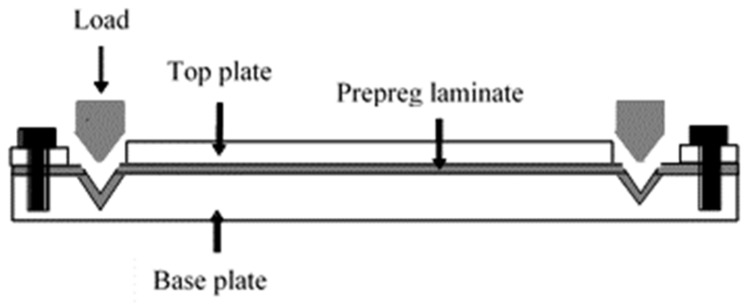
V-slot mechanical fastener [6].

The V-slot consists of an aluminium plate with V-shaped slots at the two ends (Figure 3). Unlike the deadweight method, the V-slot fastening method can be used to prestress laminates because it can accommodate material with a large surface area [33]. Prepreg laminates are prestressed by laying them up between the plates, and both ends of the material are fastened using the V-shaped bar to the slot and finally cure in an autoclave or hot press. The major drawback to using this method is a tendency to fracture when brittle fibres are being prestressed due to twisting when fastened and a difficulty in determining the prestressing level [9]. Moreover, fibre fracture can lead to a non-uniform distribution of stresses in the composite [34].

#### 3.1.3. Jack Prestress Rig Method

**Figure 4:**
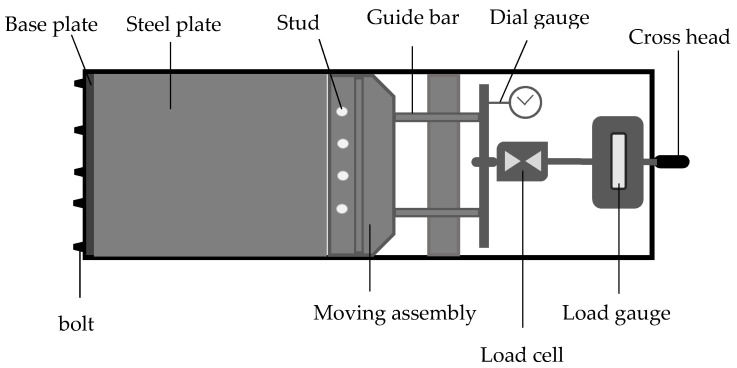
Jack prestress rig assembly.

The Jack prestress rig method was presented by Abdullah and Hassan [35]. They studied the effect of varying prestressing levels on the strength of a carbon-fibre-reinforced composite laminate. The setup consists of a flat prestress surface connected to a pulling jack. The two ends of the fibre laminate were fixed to the base plate (2) and moving assembly (4), respectively (Figure 4). The tensile force needed to produce the required prestressing level was applied through the jack and the composite was cured in an oven. However, the assembly is designed to have a free movement which might cause fibre waviness during curing.

#### 3.1.4. Filament Winding

**Figure 5:**
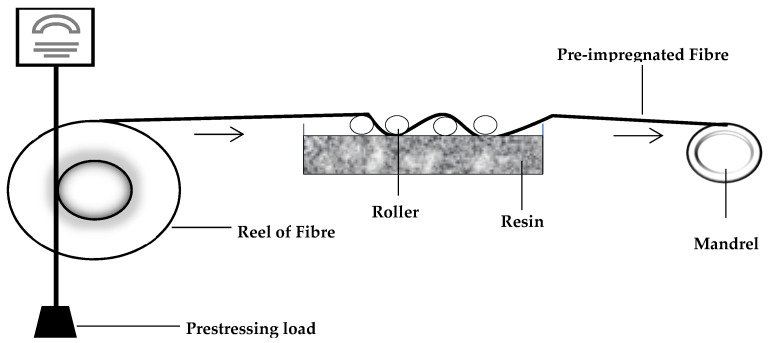
Schematic representation of the filament winding technique.

In this technique, fibres roving are pulled with a known amount of load over a liquid resin bath and then warped on a mandrel to produce a composite material (Figure 5). Composites with different prestressing levels can be made using this technique by applying varying pre-load levels [36,37]. Following the winding step, the composite is cured in an oven or autoclave or by exposing it to infrared radiation [38]. Products from this technique can vary in complexity from a simple pipe to an aeroplane fuselage [39]. The primary benefit of filament winding is the ability to use automation and robots in performing operations [40]. The greatest drawback is the difficulty in obtaining an equal fibre volume fraction due to compression and loss of resin when each layer of the composite is being warped unto the mandrel [36]. Moreover, the mandrel stiffness affects the fibre pretension; therefore, it is not easy to maintain a constant load throughout the process [9]

#### 3.1.5. Biaxial Loading Frame and Fibre Stretching Rig

**Figure 6:**
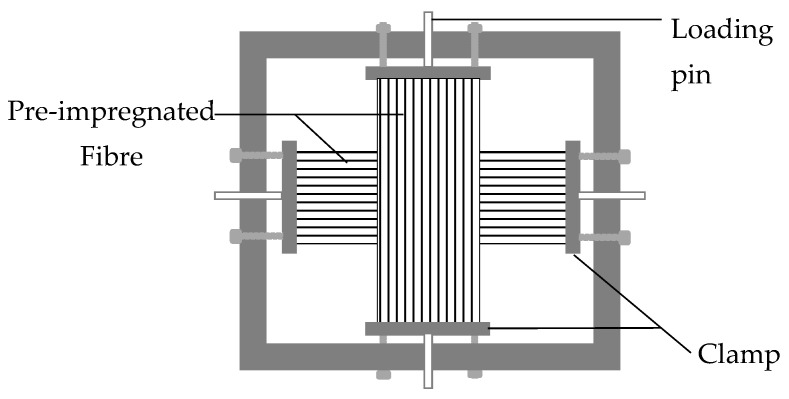
Schematic representation of biaxial loading frame.

Jevons, Fernando and Kalsi [41] studied the effect of prestressing on the low-velocity impact performance of glass fibre composites using a biaxial loading frame (Figure 6). The setup is made of a C-channel consisting of four clamps joined to the frame by bolts. Prestressing levels are applied through a loading pin by making use of a mechanical test machine. The prestressed composite is clamped and tightened with a bolt to the frame, vacuum-bagged and cured in an autoclave [42]. As a result of the sharp edge of the frame, it is difficult to prepare the assembly for vacuum-bagging [43]. Moreover, maintaining a constant level of prestressing by using the tensile machine is not easy due to the limited amount of accessible space between the fixed and moveable jaws needed to place the prestressing frame inside the device [36].

**Figure 7:**
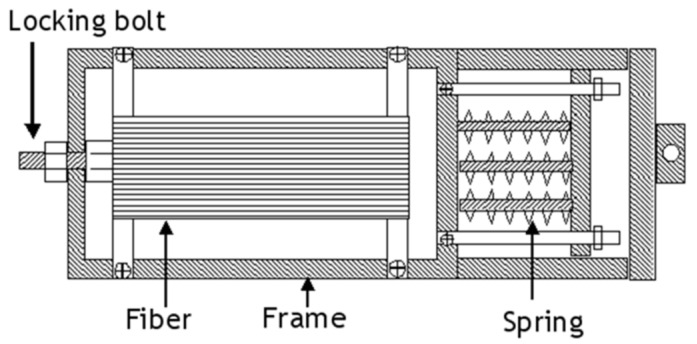
Schematic representation of a fibre-prestressing rig used by Zhao and Cameron.

Zhao and Cameron, [44] utilised a fibre-prestressing rig similar to the biaxial loading frame to prestress glass fibre-reinforced polypropylene. The fibres are wounded on the frame and preloaded using the tensile testing machine, and then the bolts are locked to achieve the required prestressing level (Figure 7).

#### 3.1.6. Horizontal Tensile Machine

**Figure 8:**
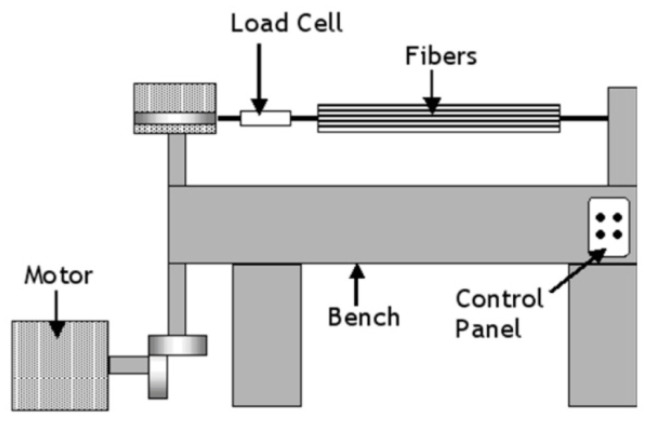
Schematic representation of a horizontal tensile machine.

**Figure 9:**
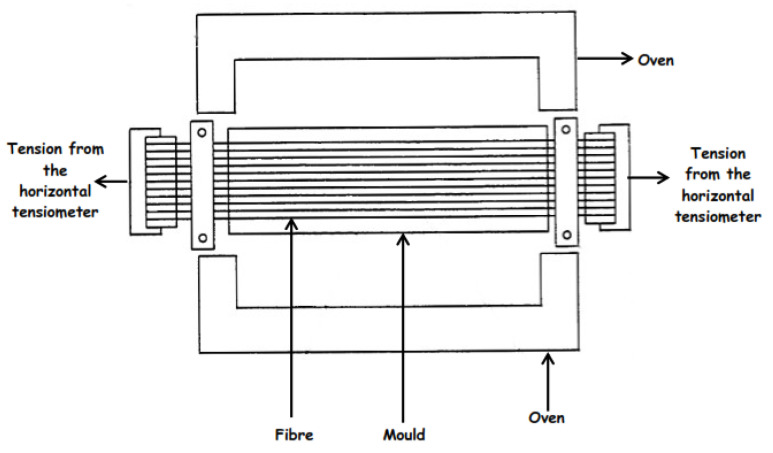
Schematic representation of fibre-prestressing using a horizontal tensile machine.

Motahhari and Cameron [45] reported a fibre-prestressing technique using a horizontal tensile machine in studying micro-residual stresses in an E-glass fibre-reinforced epoxy polymer composite. The fibres were wound on two different grips before being transferred to a horizontal tensile machine (Figure 8) that provided applied forces, and then the epoxy resin was added directly to the fibre and curing was carried out in an oven (Figure 9). The principle seems simple, but it is challenging to maintain a uniform temperature profile. Furthermore, vacuum-bagging for autoclave curing will also be difficult due to the complex assembly.

#### 3.1.7. Hydraulic Prestressing Rig

**Figure 10:**
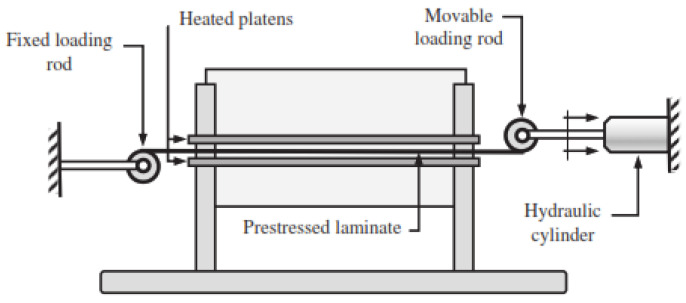
Schematic representation of the hydraulic prestressing rig [46].

Tuttle, Koehler and Keren [46] proposed a technique for fibre-prestressing using the hydraulic-cylinder-based prestressing rig shown in Figure 10. The machine consists of a movable rod connected to the hydraulic cylinder and a fixed loading rod at the other end. The prepreg was rolled around the movable rod while the tension was applied through the hydraulic cylinder that also controls the prestressing level. The technique allows the tension load to be applied only in one direction [35].

#### 3.1.8. Flatbed Prestressing Rig

**Figure 11:**
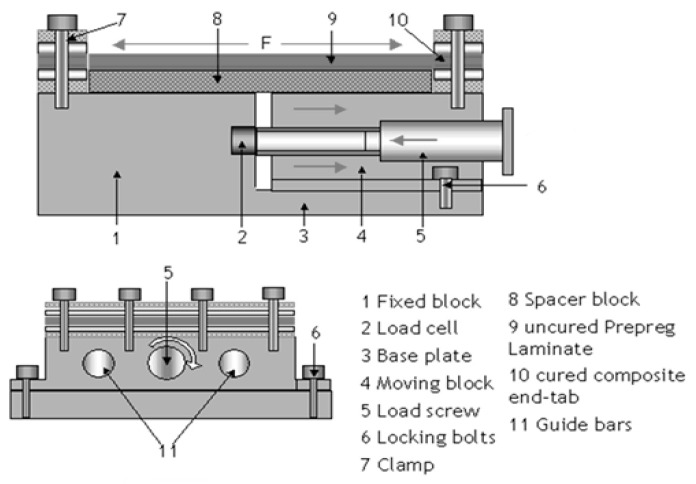
Schematic representation of the flatbed prestressing rig [42].

Krishnamurthy [35] prestressed E-glass fibre/Epoxy laminates using the flatbed prestressing rig shown in Figure 11. The laminates’ edges were cured by hot press and fixed to the rig by clamping with bolts. The loaded screw provided the tension while the laminate prestressing level was measured by the load cell (up to 150 MPa) and the autoclave was used for curing the laminate.

Fibre misalignments were identified close to the edge of the laminates due to the temperature variation between the hot press cure edge of the laminates and the remaining parts that were being cured in an autoclave [35]. The results of the requirement to subject the composite material to load throughout the curing process (EPPMC) provide the basis for the design of various prestressing techniques for a specific product geometry. Generally, EPPMC techniques can be applied to pre-impregnated fibre and both synthetic and natural fibres can be used. However, they are limited to continuous fibres, and there are constraints related to the product geometry [34].

### 3.2. Viscoelastically Prestressed Polymer Matrix Composite (VPPMC)

The technique involves using polymeric fibres to generate compressive stress in a composite material through the viscoelastic recovery process [47,48]. The fibres are prestressed by applying a load over a while to induce creep. After removal of the load, the fibres undergo a time-dependent elastic recovery. When the prestressed fibres are embedded in the polymer matrix and subjected to curing, the elastic recovery with the surrounding polymer matrix continues, thereby imparting compressive stresses that counterbalance the tensile residual stresses generated in the composite matrix [20]. Because the fibre stretching and moulding operations can be carried out independently, the technique offers flexibility in the geometry of the manufactured composite [49]. Based on previous studies, VPPMC can be classified into cellulose-fibre-based VPPMC (CFVPPMC) and synthetic polymeric fibre-based VPPMC (SPFVPPMC). CFVPPMC contain prestressed cellulose fibres predominantly from natural plants such as kenaf, sisal, flax, bamboo, jute, wheat straw, ramie and eucalyptus. Natural fibres are biodegradable and cheap and have a low density. Table 1 show physicomechnical properties of some natural and synthetic fibres commonly used in PMC.

Cui et al. [50] studied the flexural characteristics of the viscoelastically prestressed bamboo-sliver-reinforced parallel strand lumber (PSL). The flexural strength increases compared to the unprestressed counterparts. A similar result was obtained by Qin and Fancey [51] for viscoelastically prestressed cellulose yarn in a polyester casting resin matrix.

Širvaitienė et al. [52] also reported an increase in the flexural performance of viscoelastically prestressed vegetable fibre in PMC. However, due to moisture absorption, inadequate knowledge about the mechanism that controls natural fibre mechanical variations and the mode of failure, there is a limitation to their usage in polymer matrix composites [53].

Furthermore, high-performance synthetic polymer fibres are designed to resist a wide range of thermal, chemical and physical stress [58]. Nylon and ultra-high molecular weight polyethylene fibre (UHMPE) are common synthetic polymeric fibres used in polymer matrix composite development. Previous studies have shown that viscoelastically prestressed UHMPE and the Nylon 6.6 fibre enhanced the mechanical properties (strength and young modulus) of the PMC compare to their unstressed counterparts [59,60].

**Figure 12:**
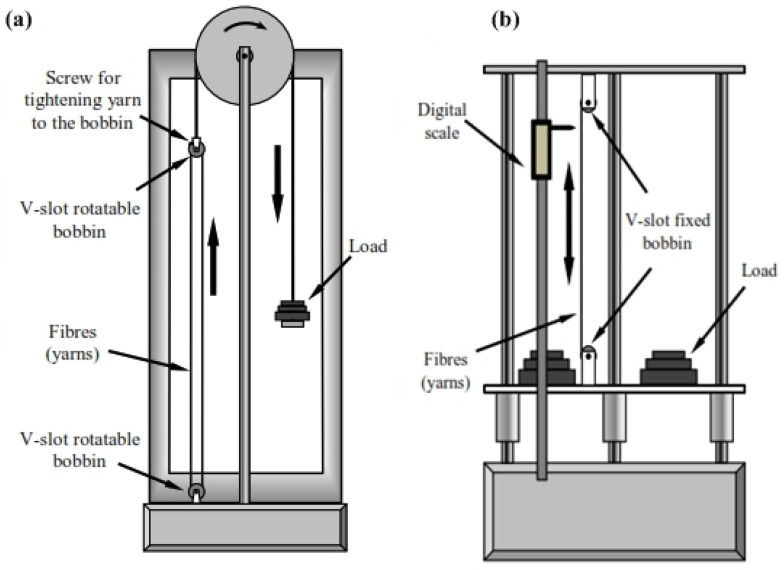
Vertical stretching rigs for VPPMC fibre-prestressing [6] Rig (**a**), Rig (**b**).

Unlike the EPPMC, in which the fibre-prestressing and curing operations are designed and carried out with the same assembly, VPPMC fibre stretching and moulding operations are decoupled therefore there is more flexibility in the fibre orientation (both continuous and short fibre can be used), distribution and material production. Many studies on VPPMC carried out fibre-prestressing using bespoke vertical stretching rigs (Figure 12). The creep and recovery strain for a small level of prestressing can be determined using the setup shown in Figure 12a, while higher level of fibre prestressing can be achieved using the rig shown in Figure 12b. The displacement gauge measures the strain and recovery rate by calculating the distance between two marks on the prestressed fibre yarn and the stretching rigs have the advantage of accommodating a wide variety of load levels required for prestressing operations [6]. On the contrary, the VPPMC technique is only applicable to fibres that have viscoelastic behaviour, and curing at elevated temperatures is not suitable to prevent a permanent deformation of the fibres [43].

### 3.3. Mechanical Properties of Prestressed PMC

Compared to other enhancing techniques, both of the two prestressing methods have shown their significance in improving the mechanical performance of composite material without increasing the mass or section dimension. Table 2 provides up-to-date findings and results of previous studies on fibre-prestressing techniques.

### 3.4. Residual Stresses Measurement

The continuous advancement in the development of composite materials has drawn more attention to prediction and measuring the distribution and sizes of residual stresses because the behaviour of composite materials is greatly influenced by these factors [72,73]. Residual stress development in the composites cannot be negligible; therefore, these can result in failure, especially when the tensile stresses generated become greater than the critical tensile strength of the material [74]. At this stage, microcracking can occur, thereby exposing the fibres to chemical and microbial attacks. Due to the nonlinear behaviour of composite material, it is difficult to measure some residual stresses that may result in material failure [75]. However, the effective measurement will reduce material failure and further enhance the research in the development of predictive control of residual stresses in composite material.

The measurement of residual stresses in the composite material can be achieved by modelling the entire production process or through the observation and measurement of the phenomena resulting in residual stress generation in a material. The latter method can be achieved through destructive and non-destructive testing. Table 3 highlights the difference between destructive and non-destructive testing techniques. The methods that are regarded as destructive typically require cutting, slitting or drilling operations to relieve the residual stresses. Afterwards, residual stresses are estimated by considering the dimensional changes that occurred. Table 4 shows some destructive and non-destructive testing methods that are commonly used to determine residual stresses in composite materials.

The layer removal method is an established method for measuring residual stresses by subjecting the material to deformation on one surface while the layers of the materials are removed from the other surface [76]. The surface of a completely equilibrated stressed component is gradually removed in layers. As a result, the portion is free of stress and a force imbalance is established throughout the system. The plate is then deformed to bring it back into balance, and the strain that results from that measurement is then utilised to quantify the amount of removed residual stress. By combining with other methods such as XRD, the layer removal method can offer information about the stress profile in a material [77]. However, this method can only assess residual stresses in materials with a simple geometry and on a macro-scale level [78].

Proposed by Mathar [79], hole drilling is commonly used in measuring residual stresses in a material. It is conducted by drilling a hole into a stressed material while the stresses relax, resulting in a change in the surrounding strain field that can be determined using a strain gauge and related to the relaxed stresses [79]. The hole-drilling method was initially designed for homogenous isotropic materials such as metals, but with a few modifications it may be used for composite, inhomogeneous and anisotropic materials, existing in both crystalline and amorphous form [80]. This method provides a more accurate measurement compared to other methods such as layer removal and ring-core methods. Moreover, the hole drilling method is less destructive because only a small portion of the central material is cut away during the deformation measurement. Likewise, it can determine the biaxial residual stresses distribution because the strain gauge is capable of identifying responses due to the biaxial surface strain of the material [81]. However, the hole-drilling method is more suitable for predicting residual stresses at the macro-scale, while it is difficult to predict at the micro-scale level of the material [82].

The hole-drilling method is divided into incremental hole drilling (IHD) and deep hole drilling (DHD). Incremental hole drilling is commonly used to measure residual stresses at varying degrees of thickness through an incremental depth drilling of the material [83]. On the contrary, deep-hole drilling involves drilling a reference hole through the material and measuring the deformation at the desired region [84]. The DHD method is faster compared to IHD but does not provide adequate insight to through-thickness residual stresses in a component, because it only provides an average residual stress over the entire depth. Thus, the IHD method is widely used compared to DHD [85]. Ghasemi, Taheri-Behrooz and Shokrieh [86] predicted non-uniform residual stresses within E-glass/Epoxy laminate composites using the incremental hole-drilling method, and the result obtained revealed that the strain at the surface decreased considerably compared to the strain released in the depth when the depth of the hole is increased. This result indicates that the residual stresses at each ply of the laminates affect the released strain of the underneath ply. A similar result was obtained by Sicot et al. [80] using carbon/epoxy cross-ply laminates, by studying the effect of increasing the drilling depth and the relative position of the strain gauge with a radius of the drilled hole. A widespread use of the hole-drilling method in determining residual stresses has led to the development of standard testing methods by The American Society for Testing and Materials (ASTM E837-20) [87].

Similarly, the ring-core method works the same way as the hole-drilling method. However, as opposed to drilling a hole, an annular groove is created, with a strain gauge rosette inserted in the middle of the groove to determine the elastic behaviour of the material [88]. The ring-core approach offers several advantages over the more popular hole-drilling technique when it comes to measuring residual stress. In the ring-core approach, more stress is released throughout the hole-drilling process, which allows for a more accurate assessment of the strain. Furthermore, because the stress concentration is reduced around the machined region, greater residual stresses can be recorded without surpassing the material’s yield stress. Additionally, the ring core method is tolerant of slight annular-hole diameter inaccuracies or eccentricity relative to the strain gauges. With this method, stress concentration effects are minimised, and it may measure stress levels close to the yield stress of the material. However, the diameter of the annular ring is relatively big, resulting in a significantly greater damage than the hole-drilling method. Because the diameter of the hole or ring determines the measuring region for residual stress, the results are also less localised.

One of the main drawbacks of the ring-core method is that it is more difficult to implement, especially when strain gauges are used, since the central wire gets in the way of the slot cutting process. Moreover, this technique has been limited to homogenous and isotropic materials [89]. The ring-core method has successfully been applied to quasi-isotropic FRP composites [90] and welded stainless steel [91]. Hence, it is obvious that the method continues to provide certain distinct advantages, especially at the micro-scale, and that additional study is required in this area to fully exploit its potentials.

The contour method is distinct among other methods used in measuring residual stresses by a relaxation mechanism because it offers detailed two-dimensional residual stress profiles that act on a plane of the material. To determine residual stresses using the contour method, the material is cut through the cross-sectional area by employing a wire Electro-Discharge Machining (EDM), and then the height maps of the cut surfaces are measured by using a laser profilometer or coordinate measuring machine. By cutting the material along the surface, residual stresses are released, resulting in deformation. The initially existent residual stresses parallel to the surface may be estimated using finite element analysis by evaluating the stresses necessary to restore the altered surface shape to a flat plane. The surface deformations are very small, and thus the EDM cutting and the surface height map measuring must be performed with very high accuracy. Furthermore, to prevent any asymmetry impacts, it is ideal to estimate the surfaces on both sides of the cut and to utilise the average surface height map. It is also feasible to make more cuts on perpendicular planes to acquire maps of the normal residual stresses along the planes. This method has been employed for measuring residual stresses in metal alloy melts [92], ion exchange glasses [93] and metal matrix composites [94]. However, the EDM cutting process can only be achieved if the material is electrically conductive, and therefore polymer-based and some fibre reinforcement, such as aramid, cannot be employed. Furthermore, the contour method can only measure uniaxial residual stresses, and it is difficult to apply the method to materials with a complex geometry [95].

The slitting method is another destructive testing method employed in measuring residual stresses. In several studies, this is also referred to as incremental slitting [96], crack compliance or compliance [97,98]. The method involves making a slit in a prestressed material and measuring the resulting deformation normal to the direction of the slit by using a strain gauge. The key advantage of using this method is its simple and non-complex procedure, its applicability to wide ranges of materials and its capability of measuring high-magnitude residual stresses. However, the slitting method application is limited to materials with reduced thickness. Therefore, it cannot adequately measure residual stresses in the materials at a macro-scale level [99].

It is standard practice in the microelectronics industry to employ Micro-Raman Spectroscopy to identify regions of mechanical stress in silicon circuit board components [100]. Chemical bonds in crystals may be studied using Raman spectroscopy, which employs light scattering to look at the vibrational energy of the chemical bonds. Raman peaks may be seen in the light scattered by the object. Any externally applied strain alters the location of these peaks. Consequently, the difference in Raman peak location between an unstressed and a stressed sample may be used to measure the applied strain. Moreover, the distribution of molecule orientations in the polymer matrix may be used to assess the strain in the amorphous polymer matrix [101]. Amorphous materials such as thermosetting polymers or glass have Raman peaks that are broad and irregular. Thermal plastics, on the other hand, have more clearly defined Raman peaks [89].

Furthermore, the X-ray diffraction method is another important technique used in measuring residual stresses in materials. This method can measure the residual stresses at the material surfaces up to a depth of 30 microns [102]. However, for deeper depth measurement, the removal of layers in the sample is required, which renders the process destructive. XRD determines the strain in the crystal lattice of a material through the examination of the change in crystal spacing. A diffracted X-ray beam may be generated by X-rays dispersed from a polycrystalline material; therefore, optimum diffracted intensities are recorded at certain angles. The diffraction plane’s inter-planar distance may be calculated from these angles by applying Bragg’s law [102]. Generally, samples with residual stresses have a spacing that is different from that of unstressed samples. Some of the advantages of using the XRD method are its simplicity and quickness in applying the process. Furthermore, the method can be applied to materials with complex shapes, and high-magnitude residual stresses at both micro and macro scales can be accurately predicted. However, the XRD method is only suitable for polycrystalline materials and the accuracy measurements can be affected by the grain size and texture [96,99]. The XRD method has reportedly been used to measure residual stresses in alloys [100,103], glass–ceramic matrix composites [104] and graphite–polymer matrix composites [105].

In addition, neutron diffraction (ND) is a method that uses neutron beam diffraction to identify deep-seated residual stresses in materials. As with X-ray diffraction, neutron diffracted beams follow Bragg’s Law, allowing stress-induced variations in atomic lattice spacing to be detected. As a result, the relative spacing differences are calibrated using a stress-free material sample to compute actual stress values. In comparison to X-rays, neutrons have the benefit of measuring residual stresses in materials at high depth and precision. The Synchrotron X-ray Method operates with the same principle as the XRD and ND discussed above.

It is a common phenomenon to employ ultrasound waves in the detection of faults in engineering materials [106], but they may also be used to quantify applied and residual loads [107]. To effectively assess stress levels in a material, it is necessary to precisely measure the time-of-flight change of an ultrasonic wave travelling between the stressed and unstressed areas of that material. To obtain an absolute measurement of stress, the time-of-flight measurement in the stressed material is compared to that in the unstressed material. The method has the advantage of measuring high-magnitude residual stresses. The measuring procedure is fast and tri-axial residual stresses can be measured [107]. However, the ultrasonic method supports only crystalline materials, is extremely sensitive to changes in the microstructure and not suitable for components with complex shapes [107].

**Table 4:** Common destructive and non-destructive test methods used in the calculation of residual stresses in composites.

Methods	Principle	Material	Shortcomings	References
Layer Removal (DT)	It monitors the elastic response of a laminate to the release of residual stresses	Ceramics Metals Polymers Composites	Additional stresses can be imparted to the test sample due to the machining of the composite surfaces. Limited to macro-scale residual stresses	[76,78]
Hole drilling (SDT)	Drilling of a hole into the stressed object releases the stresses, leading to changes in the surrounding strain field that may be measured and related to the relaxed stresses.	Ceramics Metals Polymers Composites	It requires several assumptions to simplify the result solution. Accurate measurement around the hole, especially in the fibre direction, is very challenging. Limited to macro-scale residual stress measurement	[80,82,108]
Ring-Core Method (SDT)	It follows a principle comparable to the hole-drilling method. However, instead of discharging residual stresses by drilling a hole and measuring the elastic reaction of the surrounding material, the ring-core method discharges stress by cutting an annular groove into the surface of a component that contains residual stress.	Metals Ceramics Polymers	Limited to homogenous and isotropic material.	[109]
Contour Method (DT)	The material is sliced through by a planar surface, releasing residual stresses across the plane. As a result, the surface experiences out-of-plane deformation, which is recorded, and the underlying residual stresses across the cut are calculated using the finite element technique.	Ceramics Metals Plastics Composites	Difficulty in measuring residual stresses close to the surface of the material. Not suited for small components.	[95]
Slitting Method (DT)	A tiny slit is cut into a prestressed sample, and the resultant deformation parallel to the slot’s direction induced by the restoration of force equilibrium is determined. The repetition of this procedure at increasing depths allows for the determination of residual stress across the component’s thickness.	Ceramics Metals Plastics Composites	Macro-scale residual stresses cannot be fully measured. Only average stress along the transverse direction (*y*-axis) can be measured.	[110]
Neutron Diffraction Method (NDT)	Raman spectroscopy employs light scattering to measure the vibrational energy of crystalline chemical bonds. The dispersed light is detected, and typical Raman peaks may be detected. Any externally imposed strain alters the position of these peaks. Consequently, a stressed and unstressed sample’s Raman peak position variations may be used to calculate the applied strain.	Metal Ceramics Composites	Resolution is limited, and residual stress changes smaller than 1 mm cannot be measured. Not suitable for amorphous materials	[111]
Raman Spectroscopy Method (NDT)	Stresses are determined by monitoring the frequency of certain luminescence peaks in comparison to those in an unstressed state.	Ceramics Polymers Composites	Limited to macro-scale residual stresses measurement.	[112]
X-ray Diffraction Method (NDT) (destructive if used for measuring depth)	When residual stress is determined using X-ray diffraction (XRD), the strain in the crystal lattice is determined and the related residual stress is calculated using the elastic constants, assuming that the relevant crystal lattice plane exhibits linear elastic deformation.	Metal Ceramics Composites	Applicable to polycrystalline materials only. The accuracy of this method is affected by the texture and grain size. Measurement is limited to the surface of the material	[113]
Synchrotron X-ray Method (NDT)	Similar to the X-ray diffraction method. However, X-rays are far more intense and have a much greater energy, and their tremendous energy allows them to penetrate much farther into materials.	Metal Ceramics Composites	Applicable to polycrystalline materials only	[114]
Ultrasonic Method (NDT)	The material is subjected to an ultrasonic (acoustic) wave, which is then detected by reflection, transmission or scattering. To determine the magnitude of stresses, the velocity of an ultrasonic wave in some modes is evaluated.	Metals Ceramics Composites	Not suitable for amorphous materials. Limited to macro-scale residual stress measurement.	[115]

DT: Destructive testing, SDT: Semi-destructive testing, NDT: Non-destructive testing.

## 4. Potential Applications and Prospects

Fibre-reinforced materials are used for many engineering applications where high mechanical properties are required. The use of carbon-fibre-reinforced polymer composites may reduce the weight of the materials up to 70% compared to steel [116]. Therefore, exploring such materials in making aeroplane and car parts provides the potential for minimising fuel consumption and carbon dioxide emission. Due to the high-impact energy absorption behaviour, prestressed fibre polymer composites can be used for ballistic protection in armours, vests and automobiles. Kevlar-based polymer matrix composites are materials used for ballistic protection [117]. It is well known that prestress induces an energy-absorbing mechanism at the fibre–polymer matrix interface [48], thereby increasing the impact absorption of the composite material.

Crashworthiness is an automobile’s capacity to protect its occupants from severe injury or death in the event of a given number of collisions. It is a quantitative assessment of a structure’s ability to protect its passengers in survivable crashes [118]. It is an essential factor that needs to be considered in selecting the material for vehicular structural assembly [119]. Material crashworthiness is characterised in terms of its energy absorption capacity (EAC). Generally, polymer composites have a high EAC compared to metals, and they can release deformation absorption energy during impact [120]. Prestressed fibres enhance the energy absorption capability of composite materials [70]. Moreover, the application of EPPMC and VPPMC technology in fibre-reinforced concrete development can enhance the resistance to crack propagation in a concrete structure. Bistable composite materials, which are often utilised in the aerodynamic control of aircraft and wind turbine blades, have drawn growing attention in recent years. They can be effectively manufactured utilising prestressed fibre technology [49]. The emerging developments will be toward alternate fibre materials, hybridisation, process optimisation and the use of a cost-effective fibre-prestressing technique.

## 5. Conclusions

The present article reviews some of the studies related to the techniques associated with fibre-prestressing in polymer matrix development. The presence of tensile residual stresses during the manufacturing process has a detrimental effect on composite materials’ performance, especially when subjected to an external load. Fibre-prestressing offers the advantage of minimising residual stress without increasing the mass or section dimension in composite materials. It involves generating compressive residual stresses in a fibre–polymer matrix interface to minimise the effect of tensile residual stresses. While fibre-prestressing can be achieved both elastically (EPPMC) and viscoelastically (VPPMC), several equipment designs have reportedly been used to achieve prestressing. Despite some limitations associated with these techniques, the influence of fibre-prestressing on polymer composite structural properties has been positive. Previous studies on prestressed composites have reported up to 60% increase in the tensile modulus, 62% increase in impact strength, 140% increase in tensile strength, 50% in the flexural modulus, 9% increase in compressive strength and an increase of more than 150% in fatigue life compare to their unprestressed composite counterparts.

Moreover, various methods have been reported for measuring residual stresses in materials. These are generally grouped into destructive and non-destructive methods. The destructive methods measure residual stresses by monitoring the response of a material to deformation, while non-destructive methods monitor the change in material structures when under the influence of residual stresses. The selection of the right method depends on the type of material (metals, plastics, ceramics, composites), material structure (Crystalline or amorphous), states of stresses, speed and measurement accuracy. Out of these methods, the hole-drilling method has been widely used due to its versatility and the availability of scientific standards for measuring residual stresses in various materials.

## Figures and Tables

**Table 1:** Physicomechanical properties of some commonly used fibres.

	Fibre	Density (kg/m^3^)	Tensile Strength (MPa)	Elongation at Break (%)	Young Modulus (GPa)	References
Natural fibres	Kenaf	1200	295–930	2.7–6.9	53	[54,55]
	Sisal	1200	507–885	1.9–3	9.4–22	[54,55]
	Flax	1380	343–1035	1.2–3	27.6	[54,55]
	Bamboo	800–1400	391–1000	2	11–30	[54,55]
	Banana	1350	529–914	3–10	8–32	[54,55]
	Wheat straw	1600	273	2.7	4.76–6.58	[54,55]
	Hemp	1350	580–1110	1.6–4.5	70	[54,55]
	Jute	1230	187–773	1.5–3.1	13–26.5	[54,55]
	Ramie	1440	400–938	2–4	61.4–128	[54,55]
	Rice straw	1650	449	2.2	1.21–1.25	[54,55]
Synthetic fibres	E-glass	2500	2000–3000	2.5	70	[56]
	Carbon	1800	4000	1.3	300	[57]
	Kevlar	1400	3600	2.7	130	[57]
	Nylon	1100	950	18	5	[57]

**Table 2:** Reviews on fibre-prestressed PMC.

Material	Prestress Technique	Research Area	Results of Findings	References
Glass fibre woven into a fabric Phenol-based formaldehyde resin	Elastically prestressing of the glass fibre using tensioning rod (EPPMC).	Assessment of the compressive and tension characteristics of the composite.	Enhancement of elastic properties up to 31% was recorded due to the straightening of the warp fibres.	[61]
Unidirectional graphite/epoxy prepreg tape	Prepreg tape was subjected to tension by bending over a steel roller (EPPMC).	Tensile and elastic modulus measurement	Up to 17% increase in tensile strength Composite elastic modulus was not affected	[62]
Unidirectional carbon fibre/epoxy composite with 60% fibre volume fraction	The load was applied to fibre before curing but the nature of assembly was not reported (EPPMC).	Thermal stress analysis of the composite.	Fibre-prestresses lessen the residual stresses in the matrix.	[63]
Unidirectional E-glass fibre/polyester resin with 56% fibre volume fraction	Deadweight (EPPMC)	Tensile properties evaluation	The tensile strength increases with an increase in the level of prestressing (60–80 MPa applied load). The maximum percentage increase in tensile strength and modulus obtained were 15% and 18%, respectively.	[31]
Carbon fibre/epoxy resin cross-ply laminate with 70% fibre volume fraction	Filament winding (EPPMC)	Modelling and experimental study of composite failure	Failure strength of the ply increased by increasing the prestress level up to 690 MPa	[64]
Graphite fibre/epoxy resin, unsymmetric cross-ply laminate with 56% fibre volume fraction	Hydraulic cylinder (EPPMC)	Examination of the tensile strength, curvature and transverse cracking	Fibre-prestressing reduced warping, curvature and transverse crack. Up to 28% increase ultimate strength	[47]
Unidirectional Nylon 6.6 fibre/polyester resin (up 3% fibre volume fraction)	Bespoke vertical stretching rig (VPPMC)	Analysis of the impact energy	Viscoelastically induced compressive stresses. Absorption of higher impact energy (25%) by the prestressed sample	[65]
E-glass fibre/epoxy resin cross-ply laminate (56% fibre volume fraction)	Biaxial loading frame (EPPMC)	Effect of low-velocity impact performance	25% increase in impact performance at low velocity due to prestressing	[41]
E-glass fibre/epoxy resin cross ply laminate (56% fibre volume fraction)	Biaxial loading frame (EPPMC)	Effect of high- and low-velocity impact performance	Improvement of impact performance at a low-level velocity	[66]
Unidirectional E-glass fibre/epoxy cross-ply laminates (58.2% fibre volume fraction)	Flatbed (EPPMC)	Tensile, fatigue life and compressive strength measurement	Improved fibre alignment, increase in resistance to onset damage due to induced compressive strength. 9% increase in tensile modulus and compressive strength at prestressing levels of 51 MPa and 80 MPa, respectively.	[35]
Unidirectional Nylon 6.6 fibre/epoxy resin (16, 28, 41) and 53% fibre volume fraction	Bespoke vertical stretching rig	Tensile strength and modulus measurement	30% and 15% tensile modulus and tensile strength, respectively.	[59]
Carbon and glass fibre/Hexcel cross-ply laminates	Flatbed (EPPMC)	Experimental and finite element analysis of bistable prestressed buckled laminate	Induction of bistable behaviour through prestressing.	[67]
Unidirectional Nylon 6.6 fibre/polyester resin (8, 12, 16% fibre volume fraction)	Bespoke vertical stretching rig (VPPMC)	Flexural properties measurement	Up to 50% increase in flexural modulus.	[60]
Unidirectional S-glass fibre/composite resins (Quixfil and Adoro) (12% fibre volume fraction)	Deadweight (EPPMC)	Flexural properties measurement	Increase in flexural strength.	[68]
Unidirectional UHMWPE fibre/polyester resin (3.6% fibre volume fraction)	Bespoke vertical stretching rig (VPPMC)	Impact properties measurement	Prestressing increases impact energy absorption (up to 40% increase in some batches).	[30]
Carbon fibre/epoxy resin (50% fibre volume fraction)	Deadweight (EPPMC)	Impact properties	Increase in strength of composite material.	[69]
Hybrid unidirectional Nylon 6.6 and Kevlar fibres/polyester	Bespoke vertical stretching rig (for Nylon alone) (VPPMC)	Impact and flexural test	33 and 40% rise in absorption energy and flexural modulus.	[30]
Unidirectional Nylon 6.6 fibre/polymer resin (fibre volume fraction 2.2%)	Bespoke vertical stretching rig (VPPMC)	Impact assessment	Impact energy absorbed increased (40%).	[70]
Flax yarn/polyester resin	Tension frame (EPPMC)	Tensile and flexural assessment	Fibre alignment enhancement. Increased tensile strength and modulus. Increase in flexural strength and modulus.	[26]
Plain weave E-glass fabric/polyester resin (16% fibre weight fraction)	Hydraulic cylinder biaxial loading frame (EPPMC)	Flexural characteristics	Up to 16% increase in flexural strength at 50 MPa optimum prestressing level	[71]
Plain weave E-glass fabric/polyester resin (11% fibre weight fraction)	Hydraulic cylinder biaxial loading frame (EPPMC)	Tensile and fatigue characteristics	Fatigue life increased up to 43% Fatigue life improvement when under low and intermediate stress fatigue load	[43]
Unidirectional E-glass fibre mats/epoxy resin	Horizontal testing machine (EPPMC)	Flexural, tensile and compression properties	Reduction in fibre waviness Increase in flexural, tensile and compressive strength	[8]
Nylon 6.6 yarn/polyester cross-ply composite	Bespoke stretching rig (VPPMC)	Impact behaviour	Up to 29% reduction in damage depth	[28]
Unidirectional E-glass fibre/epoxy resin (10% fibre volume fraction)	Deadweight method (EPPMC)	Tensile properties	Increase in maximum strength, percentage elongation and rupture strength by 38.5%, 45.57% and 106.2%, respectively	[32]

**Table 3:** Difference between destructive and non-destructive testing.

Destructive Testing (DT)	Non-Destructive Testing (NDT)
Part of the materials is removed or damaged.	Testing can be done without removing or damaging the material.
Testing cannot be repeated on the same specimen.	Testing can be repeated on the same specimen.
Residual stress measurement is limited to a small area of the material sample.	Residual stresses can be measured within a large surface (e.g., laminate).
Global residual stresses distribution along the plies in a composite can be measured.	They cannot estimate global residual stress distributions along with composite plies.

## Data Availability

The data presented in this study are available on request from the corresponding author.
